# A TWO-YEAR RCT EXAMINING SPEED OF PROCESSING TRAINING ON EVERYDAY FUNCTIONING IN ADULTS WITH HAND IN THE US DEEP SOUTH

**DOI:** 10.1093/geroni/igad104.1687

**Published:** 2023-12-21

**Authors:** David Vance, Pariya Fazeli, Andres Azuero, Sarah Khalidi, Jennifer Frank, Virgina Wadely, James Raper, Caitlin Pope

**Affiliations:** University of Alabama at Birmingham, Birmingham, Alabama, United States; University of Alabama-Birmingham, Birmingham, Alabama, United States; University of Alabama at Birmingham, Birmingham, Alabama, United States; University of Alabama-Birmingham, Birmingham, Alabama, United States; University of Alabama-Birmingham, Birmingham, Alabama, United States; University of Alabama-Birmingham, Birmingham, Alabama, United States; University of Alabama-Birmingham, Birmingham, Alabama, United States; University of Kentucky, Lexington, Kentucky, United States

## Abstract

Approximately 44% of people living with HIV (PLWH) experience HIV-associated neurocognitive disorders (HAND). Cognitive training approaches, such as speed of processing (SOP) training, may reduce the detrimental impact of HAND on everyday functioning. In this experimental design study, 216 participants 40 and older with HAND or borderline HAND (82% Black) were randomized to one of three groups: 1) 10 hours of SOP training (n=70); 2) 20 hours of SOP training (n=73), or 3) 10 hours of Internet Navigation Control Training (a contact control group; n=73). Participants completed several everyday functioning measures at baseline, posttest, and year 1 and year 2 follow ups. Everyday functioning measures included: 1) Modified Lawton and Brody Activities of Daily Living (ADL) Questionnaire; 2) Timed Instrumental Activities of Daily Living (TIADL) Test; 3) Patient’s Assessment of Own Functioning (PAOFI); 4) Medication Adherence Questionnaire (MAQ); and 5) Medication Adherence Visual Analog Scale (VAS). Linear mixed-effect models and generalized estimating equation models were fitted to estimate between group differences at all follow-up time points. At follow-up timepoints, those in the 10-hour and 20-hour training groups performed better on medication adherence measures (MAQ and VAS) than those in the control group, with effects (Cohen’s d) ranging from 0.13 to 0.41 for MAQ and 0.02 to 0.43 for VAS. In conclusion, SOP training improved some indicators of everyday functioning, specifically medication adherence; however, the therapeutic effects diminished over time. This is an interesting finding given the importance of medication adherence in viral suppression and overall survival in PLWH.

